# Aspiring to clinical significance: Insights from developing and evaluating a machine learning model to predict emergency department return visit admissions

**DOI:** 10.1371/journal.pdig.0000606

**Published:** 2024-09-27

**Authors:** Yiye Zhang, Yufang Huang, Anthony Rosen, Lynn G. Jiang, Matthew McCarty, Arindam RoyChoudhury, Jin Ho Han, Adam Wright, Jessica S. Ancker, Peter AD Steel

**Affiliations:** 1 Department of Population Health Sciences, Weill Cornell Medicine, New York, New York, United States of America; 2 Department of Emergency Medicine, Weill Cornell Medicine, New York, New York, United States of America; 3 Department of Emergency Medicine, Vanderbilt University Medical Center, Nashville, Tennessee, United States of America; 4 Geriatric Research, Education, and Clinical Center, Tennessee Valley Healthcare Center, Nashville, Tennessee, United States of America; 5 Department of Biomedical Informatics, Vanderbilt University Medical Center, Nashville, Tennessee, United States of America; King George’s Medical University, INDIA

## Abstract

Return visit admissions (RVA), which are instances where patients discharged from the emergency department (ED) rapidly return and require hospital admission, have been associated with quality issues and adverse outcomes. We developed and validated a machine learning model to predict 72-hour RVA using electronic health records (EHR) data. Study data were extracted from EHR data in 2019 from three urban EDs. The development and independent validation datasets included 62,154 patients from two EDs and 73,453 patients from one ED, respectively. Multiple machine learning algorithms were evaluated, including deep significance clustering (DICE), regularized logistic regression (LR), Gradient Boosting Decision Tree, and XGBoost. These machine learning models were also compared against an existing clinical risk score. To support clinical actionability, clinician investigators conducted manual chart reviews of the cases identified by the model. Chart reviews categorized predicted cases across index ED discharge diagnosis and RVA root cause classifications. The best-performing model achieved an AUC of 0.87 in the development site (test set) and 0.75 in the independent validation set. The model, which combined DICE and LR, boosted predictive performance while providing well-defined features. The model was relatively robust to sensitivity analyses regarding performance across age, race, and by varying predictor availability but less robust across diagnostic groups. Clinician examination demonstrated discrete model performance characteristics within clinical subtypes of RVA. This machine learning model demonstrated a strong predictive performance for 72- RVA. Despite the limited clinical actionability potentially due to model complexity, the rarity of the outcome, and variable relevance, the clinical examination offered guidance on further variable inclusion for enhanced predictive accuracy and actionability.

## Author summary

This study developed a model to predict emergency department (ED) return visit admissions (RVA) which are clinical events in which patients discharged from the ED rapidly return and require hospital admission. RVA are multifactorial but have been associated with preventable adverse outcomes. We developed a predictive model by evaluating several machine learning techniques and compared this to an existing clinical score. The model drew data from electronic health records at three urban EDs. Clinicians conducted a clinical evaluation of the model input and output through manual chart reviews. The model was found to be predictive and fairly generalizable but lacked clinical actionability. Findings will inform future model development process by ensuring that we include variables are that predictive and explainable to clinicians in a way that leads to actionability.

## Introduction

Emergency department (ED) return visit admissions (RVA) refer to instances where patients discharged from the ED return or “bounce back” to the ED within a short period and subsequently require hospital admission. RVA within 72 hours of initial ED visit occur following over half a million US ED visits annually. [1] Prior work has shown RVA to be associated with adverse outcomes, such as increases in mortality and ICU admission, higher needs for surgical interventions, and longer hospitalizations. [2–6] These outcomes are unique to RVA and have not been identified in all ED return visits, a much more frequent, lower acuity outcome. [7] More recently, RVA case reviews have been utilized to identify quality improvement opportunities, as well as delayed diagnoses in specific disease groups. [8–11] However, the etiology of RVA are multifactorial, inclusive of complex patient, system, and physician factors such as shared decision-making, patient non-compliance, adverse social conditions, disease recurrence, inadequate outpatient care, unforeseen rapid disease progression, and suboptimal management by the ED care team. [8,12–15] Known risk factors of RVA include older age, severity of illness, renal disease, congestive heart failure, vital signs during ED stay, medical error, as well as social factors such as ambulatory status, family support and insurance status. [2,16]

The lack of clinical tools to prevent RVA has been found as a crucial barrier and is compounded by two factors: the technical problem of RVA prediction and the translation problem of converting the prediction into actionable tools. The identification of patients at high risk for acute post-ED adverse events remains a complex endeavor, hindered by the combinatorial explosion of potentially relevant risk factors of RVA. [17] Information generated from the ED is highly heterogenous, including a variety of information from ED presentation to ED discharge, in addition to medical history, prior visits, and social determinants of health (SDoH). The principal conditions for ED presentations also vary in disease and disease severity across patient populations. [1,18] The episodic ED care model often results in a high degree of missing and potentially erroneous data. These characteristics render the RVA prediction a technically challenging problem that calls for innovative analytical techniques tailored to heterogeneous ED data. Moreover, beyond accurate prediction, the actionable use of machine learning outputs to the care team is paramount. In this study, we define the actionability of RVA prediction as the interpretability of the models and the straightforward application of their predictors in initiating concrete clinical interventions to mitigate the risk. The ability of a model to not only signal risk but also provide actionable insights into both the etiology of risk and potential interventions is essential for translating predictive power into clinical impact.

### Objectives

This study aims to use machine learning to develop a predictive model for RVA. We studied the predictive robustness of our approach and its practical application in the ED setting, with the eventual goal of enhancing patient’s health trajectories and mitigating the occurrence of RVA. This paper delves into the specifics of the machine learning training process using two EDs’ EHR data, and our process for retrospective validation at an independent hospital. A clinical evaluation was also performed to both examine model performance across different clinical scenarios [19], and support the future development of a clinical response protocol in which the machine learning model will be included.

## Results

**Fig 1:**
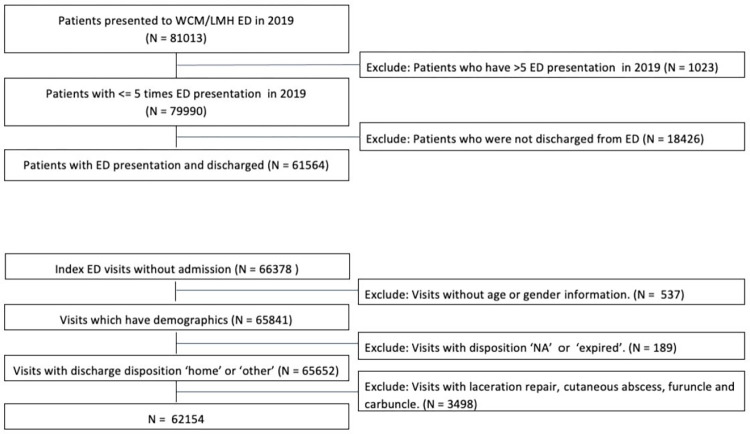
Inclusion Criteria (Development Site). WCM: Weill Cornell Medicine. LMH: Lower Manhattan Hospital.

A total of 61,564 qualifying patients who had 62,154 ED visits in 2019 were included in the study in the development site. Using the same inclusion and exclusion criteria, we included an independent validation set of 73,453 patients who presented to another urban ED in 2019. Fig 1 describes the inclusion criteria. Between the development and validation sites, there is significant difference in the racial distributions, age, sex, insurance types, and ability to speak English among patients (p-value <0.01). However, there is no statistically significant difference in the proportions of 72-hour RVA cases between the two ED sites. Table 1 displays the patient characteristics in the study cohort. RVA within 72 hours were experienced by 508 (0.82%) of ED treat-and-release patients in the development site, and 571 (0.78%) in the validation site. There is a statistically significant difference in the racial distributions and insurance types between the total population and those who had 72-hr RVA in the development and validation sites (p-value <0.05). Between the total population and RVA cases, there was no statistically significant difference in sex in the development site, but a significant difference was observed in the validation sites. No difference in English language or age was observed between the total population and RVA cases either in the development or validation sets. Table 2 describes the common diagnoses defined in ICD-10 observed in RVA, which provide a clinical view of factors related to the RVA.

**Table 1:** Patient Demographics.

		Development Site		Validation Site	
		Total (N = 62154)	72-hour RVA (N = 508, 0.82%)	Total (N = 73453)	72-hour RVA (N = 571, 0.78%)
**Race**	**White (%)**	25358 (40.80%)	258 (50.79%)	15245 (20.75%)	157 (27.5%)
	**Black (%)**	13288 (21.38%)	79 (15.55%)	8317 (11.32%)	69 (12.08%)
	**Other (%)**	14630 (23.54%)	113 (22.24%)	33563 (45.69%)	207 (36.25%)
	**Asian (%)**	4715 (7.59%)	50 (9.84%)	15014 (20.44%)	136 (23.82%)
	**Unknown (%)**	4163 (6.70%)	8 (1.57%)	1314 (1.79%)	2 (0.35%)
**Language**	**English-speaking (%)**	54031 (86.93%)	443 (87.20%)	43468 (59.18%)	347 (60.77%)
**Sex**	**Female (%)**	34945 (56.22%)	285 (56.10%)	39633 (53.96%)	281 (49.21%)
**Age (sd)**		47.7 (18.55)	55.7 (20.51)	37.67 (24.73)	46.83 (28.92)
**Insurance**	**Commercial (%)**	29182 (46.95%)	187 (36.81%)	23037 (31.36%)	129 (22.59%)
	**Medicaid (%)**	14992 (24.12%)	114 (22.44%)	32861 (44.74%)	244 (42.73%)
	**Medicare (%)**	12344 (19.86%)	183 (36.02%)	11278 (15.35%)	172 (30.12%)
	**Self-pay (%)**	5569 (8.96%)	23 (4.53%)	6266 (8.53%)	25 (4.38%)

**Table 2:** Characteristics of RVA visits.

RVA Disposition	72-hour (N = 508, 0.8%)	
	N (%)	ICD-10-CM diagnoses (N)
Home	379 (74.61%)	Other sepsis (19), Cellulitis and acute lymphangitis (15), Other disorders of urinary system (14),
Other	54 (10.63%)	Other sepsis (4), Zoster [herpes zoster] (3), Malignant neoplasm of colon (2), Fracture of forearm (2), Fracture of femur (2), Other disorders of urinary system (2), Type 2 diabetes mellitus (2)
Left AMA	71 (14.0%)	NA (13) Pain in throat and chest (5) Abdominal and pelvic pain (4) Find of drugs and other substances, not normally found in blood (3)
Deceased	4 (0.79%)	Other sepsis (1), Atheroembolism (1), Intracranial and intraspinal phlebitis and thrombophlebitis (1), Intracranial injury (1)

### Prediction performance

Experiment results on the 72-hr RVA are shown in Table 3 sorted by AUC values achieved in each experiment. The AUC ranged between 0.611 to 0.874 for 72-hour RVA. The highest AUC is 0.874 (95% CI: 0.842–0.907s) achieved by DICE+LR+FFS (applying LR to DICE cluster membership and variables selected by FFS), moderately outperforming LR+FFS without DICE (applying LR to variables selected by FFS). Table 3 also lists the positive likelihood ratio, which is the likelihood that a positive prediction will occur in a patient with RVA compared to the likelihood that a positive prediction would be expected in a patient without RVA. LR+FFS has the highest ratio, followed by DICE + GBDT and XGboost.

**Table 3:** 72-hr RVA Predictive Performance.

Site	Model	AUC	Sensitivity	Specificity	Positive Likelihood Ratio
**Development (test set)**	DICE + LR + FFS	0.874	1.000	0.559	2.268
	LR + FFS	0.872	0.754	0.806	3.887
	XGboost	0.829	0.600	0.826	3.448
	DICE + GBDT	0.828	0.708	0.802	3.576
	DICE + XGboost	0.828	0.623	0.740	2.396
	LR	0.793	0.615	0.785	2.860
	DICE + LR	0.783	0.523	0.876	4.218
	GBDT	0.759	0.692	0.719	2.463
	Risk score prediction (modified score)	0.704	0.692	0.528	1.466
	Risk score prediction	0.611	0.446	0.727	1.634
**Validation**	DICE + LR + FFS	0.75	0.37	0.93	5.52

We applied the model without any modification to the independent validation set. The same inclusion criteria and data processing were performed. The model performance achieved an AUC of 0.75, sensitivity 0.37, specificity of 0.93, and positive likelihood ratio of 5.52.

**Fig 2:**
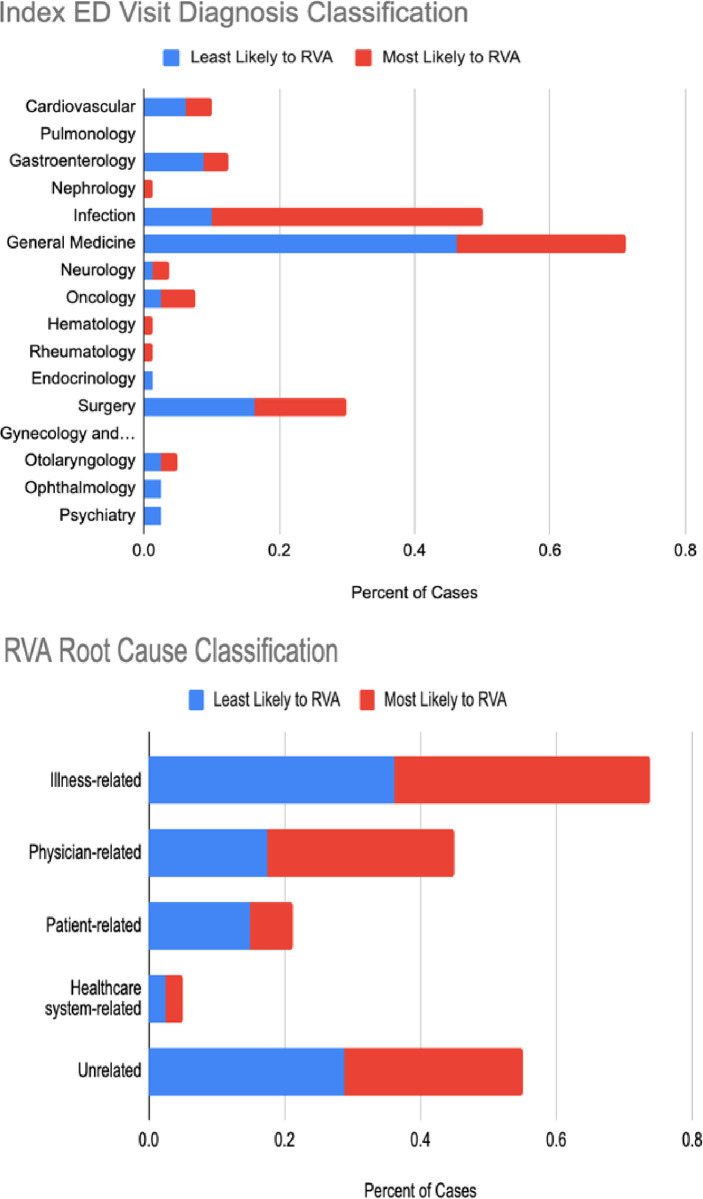
SHAP visualization of 72-hour RVA features.

We observed variables that featured predominantly in a positive prediction of RVA across individual patients. These include membership in the high-risk DICE cluster, orders for patient navigator consults, use of opiate, orders for blood culture tests, and lactate ringers. To provide information about the high- and low-risk DICE clusters, we evaluated the risk ratio of 72-hr RVA across clusters. A risk ratio is a measure used to determine the relative risk of a certain outcome occurring in one group compared to another. The risk ratio between higher- and lower-risk clusters is 2.96 and 4.51 in the training and test sets, respectively. This indicates that the higher-risk clusters found by DICE have a greater likelihood of 72-hr RVA (4.51 times in the test set) occurring compared to the lower-risk clusters. In addition, Fig 2 lists 20 model variables selected by FFS, ordered by their variable importance according to the mean absolute SHAP values obtained in the test set. The most important variables in predicting 72-hour RVA included the probability to belong in high- and low-risk clusters derived by DICE, pain in throat and chest, Basophil count, consult order for patient navigators.

### Sensitivity and bias analyses

We conducted additional analyses in the development site to better evaluate the model performance and bias. The AUC for 72-hour for White, Black, Asian, and Other racial subgroups were 0.881 (95% CI: 0.83–0.93), 0.922 (95% CI: 0.87–0.97), 0.825 (95% CI: 0.73–0.92), and 0.833 (95% CI: 0.75–0.92), respectively. The sensitivity analysis included testing on patients aged 65 years old or above, and across relevant disease categories. Removing variables providing information around discharge in the prediction decreased AUC by 3%. Limiting the analysis to patients 65 and older decreased AUC by 1.1%. The decrease in AUC was the largest among patients with HF (AUC = 0.58). The AUC increased for UTI patients in predicting 72-hr RVA (AUC = 0.94).

### Clinical evaluation

**Fig 3:**
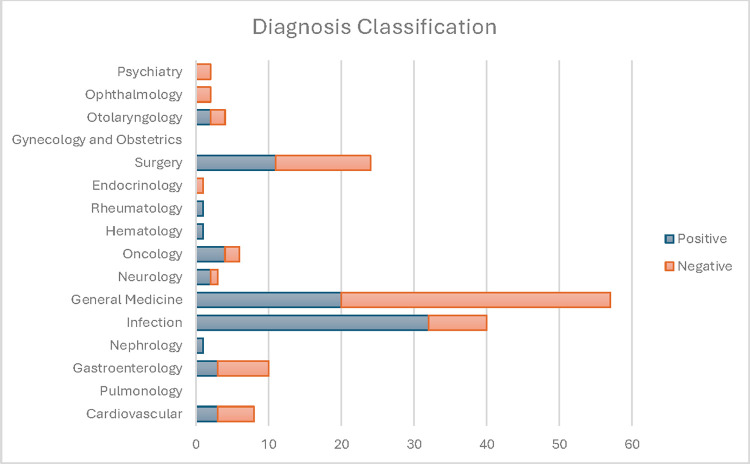
RVA Evaluated by Index ED Visit Diagnosis Classification. Positive: model predicted RVA; Negative: model missed RVA.

**Fig 4:**
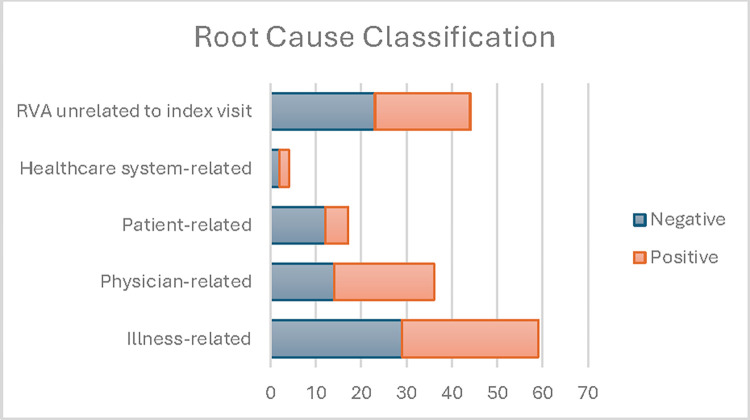
RVA evaluated by index ED visit root cause classification. Positive: model predicted RVA; Negative: model missed RVA.

Figs 3 and 4 display the clinical evaluation results. We selected the 160 patients predicted to have the 80 highest and 80 lowest risks of RVA. The red and blue colors indicate a positive and a negative prediction, respectively. Infectious diseases related RVA were more commonly identified by the model (40%) compared to those missed by the model (11%), with a significant difference (p<0.001). General Medicine diagnoses were more prevalent in RVA missed by the model (46.3%) compared to those identified by the model (33.3%, p = 0.008). Illness-related causes such as disease progression and recurrent disease were common in both model-identified and missed RVAs. Physician-related causes were noted in 27.5% of model-identified and 17.5% of model-missed RVAs, with no significant difference in model performance across root cause categories. Arbitration by the third reviewer was necessary in under 5% of the cases.

## Discussion

This paper describes our effort to develop and validate a predictive model for RVA that achieves both predictive performance as well as interpretability. The highest predictive performance as measured by AUC resulted from the use of DICE, a novel predictive clustering algorithm which identified clusters of patients based on their risk of RVA and patient similarity. DICE was especially successful in identifying representations and clusters concerning the 72-hour RVA outcome, as observed in the risk ratio of 4.5 between clusters. While there is limited prior work on the prediction of RVA, [20] our performance in terms of AUC is strong as demonstrated in our study data. We subsequently used the cluster membership to train a binary classifier with additional variables selected from the EHR to boost interpretability. Predictors in the classifier were consistent with previous studies, including insurance payor status, higher disease acuity, ED LOS, and chronic conditions such as HF. [12,21,22] On the other hand, our EHR-derived predictors did not include known predictors related to cognitive impairment and functional status. [23]

Our quantitative analysis of model performance across age and disease categories exhibited variability, which may be explained by the constraints of the clinical evaluation of ED patients. Accurate prediction of RVA is reliant on input that will represent a conceptual “data prodrome” for the RVA event, using data available by the end of the index ED visit. This prerequisite for performance could bias the model towards clinical scenarios and patients that produce, or are represented by, a data prodrome of distinguishable EHR variables. This hypothesis is supported by the superior predictive performance in the infectious disease group observed in the clinical examination, clinical scenarios (e.g.: urinary tract infection) which may more consistently produce variable input data patterns. Slightly lower performance of the model in older adults complements prior work describing a loss of physiological reactivity with aging, as well as afebrile infections and other differences in disease expression that may limit the formation of a data prodrome during the initial ED visit to suggest a high likelihood of RVA.[24, 25] While the results of the model examination deliver some transparency into current model performance across differing clinical scenarios, the findings can also provide directionality to future exploration of additional input variables to improve performance in granular cases such as predicting RVA-related patient non-compliance with the post-ED care plan. Future research will also prospectively evaluate the effectiveness of the machine learning model, including comparison to real-time physician judgement to promote our understanding of the model’s impact compared.

Further, our “data prodrome” hypothesis offers promise for the clinical value of the model, since prior work describes the majority of RVA events occur when the care team misjudge the disease trajectory and the likelihood of disease progression, as evidenced in the successful implementation of machine learning-based CDS for early sepsis recognition.[26–28] Similarly, high model performance in RVA determined to be the result of physician-related factors, (e.g., diagnostic error), may also be explained by under-interpreted EHR data (e.g.: abnormal lab values, or vital signs).[26,27] Despite these correlations, model output into two distinct phenotypes–high risk and low risk for RVA–is unlikely sufficient clinical decision support (CDS) to inform ED care teams of meaningful next steps. Identifying clinical subgroups of RVA with discrete model performance characteristics is likely the first step to developing output beyond the binary, towards a goal of output that can guide meaningful next steps in patient care. ED disposition decisions are often more complex than a simple dichotomous decision of admit versus discharge. Despite the recognition of patient vulnerability, hospital admission on an initial ED visit may not be the best solution for many patients at risk of RVA, who could alternatively benefit from individualized post-ED care management and transitions-of-care interventions.[29,30] Further, RVA etiology is multifactorial; the appropriate mitigating actions are likely dramatically different in the setting of an anticipated RVA due a patient’s non-compliance with their post ED care plan, compared to a missed diagnosis of a critical illness that warranted admission at the initial ED visit. The complexity of RVA etiology requires a CDS design that will inform the variable downstream clinical workflows to mitigate risk. Thus, future work will focus on further developing model output, such as multiple outcome-aware clusters from DICE, that can inform next steps in individual patient’s care, a transformative approach shown to improve patient outcomes. [31,32]

This study has several limitations. Due to the use of EHR data within a single health system, we likely missed patients who had RVA at hospitals outside the study sites. [33] Future study will leverage multi-site data or insurance claims data to mitigate this limitation. A technical challenge for RVA prediction is the low outcome rate leading to moderate positive likelihood ratio by the model. False positives may lead to alert fatigue and wasted resources, whereas false negatives may potentially miss opportunities to identify and intervene on patients at risk of adverse events. We tackled the data imbalance issue with several approaches, including sampling (oversampling of the minority class), learning similarities from patients admitted from their index ED visits, changing the neural network architecture including hyperparameter tuning. Specifically, we attempted to capture and learn from the nuanced similarities among patients admitted from their index ED visits so that our model could better generalize across the outcome of RVA. In training DICE, we experimented with different architectures and layers that are known to perform well under imbalanced data conditions. We ultimately observed the best performance through hyperparameter tuning to find the optimal set of parameters that allowed our model to better predict RVA. Future research can examine methods such as contrastive learning[34] and a cascade approach to further filter false positives.[35] Relatedly, while DICE was intended to identify clusters that have similar risk level but differing patient characteristics, in this study, we found the best predictive performance resulted from 2-clusters: one high-risk and one low-risk. This may be due to the rarity of the outcome and small sample size. Future work will increase sample size to further study the derived clusters centered on RVA.

In addition, our evaluation of the trained model in the validation site observed a decrease in performance by an AUC of 0.12. The decrease may be attributed to change in patient population and clinical practice. Since the model’s input variables, particularly orders, are influenced heavily by the workflow, a change in hospital and thus a change in workflow may affect the model performance. Since this study used only one year worth of data, in future model development phases we will use larger datasets, inclusive of unseen populations and clinical environments, to further develop and evaluate the model’s generalizability and evaluate its stability against distributional shift by using datasets from time periods such as the Covid pandemic era of 2020–21. [36]

In summary, our machine learning approach using two hospitals’ EHR data predicted 72hr ED RVA, outperforming a single heuristic RVA clinical risk score. The success potentially derives from utilizing a diverse array of variables that contribute to precise risk identification, including the patient’s clinical profile, complexity of the ED presentation itself, and influential social determinants. [37] This study highlights the potential value of predictive analytics in emergency medicine: the development of automated, precision screening and prediction tools in a uniquely challenging environment, where traditional manual CDS tools have been inconsistently integrated into clinical practice. [38–45] We also identified crucial area for improvement around clinical significance from limited direct actionability from the model output. Additional methodological development is warranted for this model to serve as EHR-based CDS for ED clinicians.

## Material and methods

The study design, and the model construction and evaluation process, are described below.

### Relevant work

Despite the potential gain in patient outcomes if RVA are prevented, the development of predictive tools tailored to RVA has been limited. [4,16,20,21,23,46] Meldon et al. developed a triage risk stratification tool (TRST) to predict the composite outcomes of return visits (RV) and hospital admissions among elder patients following their ED discharge. [23] McCusker et al. developed the identification of seniors at risk (ISAR) which is mainly aimed to predict all ED return visits (RV), a much more frequent, lower acuity outcome compared to RVA. [7] Both screening tools are intended for manual screening and consist of questions that rely on self-disclosure and direct assessments by healthcare professionals. Recent work has taken more predictive model approaches to automate screening without requiring manual assessments. Gabayan et al. developed a risk score to predict 7-day RVA derived from multivariate logistic regression among older adults aged 65 and above. [4] Hong et al. and Hao et al. developed machine learning models to predict 72-hour, 9-day, and 30-day RV using EHR data. [47,48] Relatedly, others have develop machine learning models to predict admission at time of ED triage and after ED evaluation. [49,50] In addition, there are technical parallels between RVA and 30-day all-cause hospital readmissions, a problem for which machine learning has demonstrated notable efficacy, particularly in adapting to the non-linear and intricate relationships between variables. [51,52]

Quality-based research has most frequently explored ED RV and RVA within 48 hours to 7-days post index visit, with 72 hours between initial to return visit being the most frequently studied and near consensus “clinically high-yield” time-period. [2,5,8,21,22,53] One large multistate study by Rising et al on RV advocated for a 9-day period to capture patients’ unmet “acute care needs”. However, this study also demonstrated that the 75% of the 9-day RV occurred within the first 72 hours, further informing this study’s target outcome. [54]

### Study design

This is a retrospective observational study using variables extracted from the EHR. Study data, including the development and validation sets, were obtained from two EDs using encounters from 2019. The development site includes one of quaternary care, urban, academic hospital, and the other of a medium-sized urban community hospital. The validation set was drawn from a medium-sized urban community hospital. The three hospitals are divergent in both geographical location and demographic population served. The outcome is defined as RVA within 72 hours since the index ED visit discharge.

### Participants

Inclusion and exclusion were applied at the patient level, and subsequently, also at the visit level. At the patient level, we included all patients presenting to the adult ED and were discharged or left against medical advice from their index ED visit. We also excluded patients who left the ED without being evaluated or seen by a healthcare provider and patients transferred to other acute care facilities. These criteria include patients discharged to home, as well as to a non-acute care facility such as a nursing home or a non-acute care facility. We excluded all patients who were admitted to the hospital from their index ED visit. Patients presenting to the psychiatric ED were removed from the study. Patients who had more than five ED visits in a year, a common definition for ED “High-Utilizers”, were also removed from the study. [55,56] High-utilizers, or patients who visit the ED with significantly higher frequency compared to the rest of the population, have been well described in the literature and frequently do not require acute hospital admission. [57] This distinct subgroup of ED patients have common multidimensional characteristics, and typically require extensive longitudinal case management to successfully impact their avoidable healthcare utilization reflective of their challenging chronic care needs. [58] High-utilizers are thus conceptually distinct and have different healthcare needs compared to the vast majority of RVA patients, who quickly bounce back to the ED and require admission due to inadequacy of their initial post-ED care plan regarding their acute care needs.

Since patients may have multiple visits, we treated each visit as an index visit to determine if the following ED visit qualifies as an RVA, the study outcome. Among the study eligible patients, to remove index visits with scheduled revisits, we excluded those encounters with diagnoses codes associated with planned return ED visits, such as laceration repairs for suture removal, and wound checks. Diagnoses associated with excluded visits are listed in S3 File.

### Data

For each patient, our study variables include information recorded from ED presentation to discharge in the index visit. These include information on socio-demographics, diagnoses, therapeutics (e.g.: medications), laboratory test orders and test results, vital signs, the frequency of imaging tests, operations and utilization data, and other clinical care variables. The socio-demographic information includes age, gender, race, marital status, preferred language, and insurance payor. Diagnoses were extracted using ICD-10-codes and additionally represented as Elixhauser comorbidity index to indicate the comorbidity burden. [59] Medications were grouped into drug classes according to MedlinePlus. [60] For vital signs, we summarized the sequential values as one-time variables: first, last, maximum, minimum, and average of all values recorded in the ED. For lab values, we used the average from the ED stay.

Proposed variables were evaluated by the clinical team prior to model construction to optimize clinical interpretability and meaningfulness. Diagnostic tests that were resulted after patients’ discharge time, variables determined clinically irrelevant (e.g.: EHR system messages), and informally *scheduled* ED return visits (e.g.: planned return visits for suture removal, wound checks) were all excluded before model construction. For diagnostic and medication orders, we included a measure for variability across patients, defined as order frequency-inverse patient frequency, which is a numerical statistic intended to reflect how important an order is to a patient in a cohort, inspired by frequency-inverse document frequency (TF-IDF). [61] Additionally, the total number of diagnostic imaging tests was summed as one variable, used as a surrogate for the complexity of emergency medical care received. Qualitative results of imaging tests were not included, as the model only uses structured information. The operations and utilization variables included disposition (e.g.: home, care facility), length of stay (LOS), frequency of ED visits in the prior year, emergency severity index (ESI), and campus location. Other clinical care variables included service consultations completed while patients were in the ED. Numeric and continuous variables from individual patients were represented as normalized vectors. All numeric variables were normalized with a mean of 0 and a standard deviation of 1.

### Ethics statement

This study has been reviewed and approved by Weill Cornell Medicine Internal Review Board (protocol number 23–08026440, https://hrp.weill.cornell.edu/irb). Due to the retrospective nature of the study, we have requested and received approval for waiver of informed consent.

### Machine learning

The algorithm used in this work, deep significance clustering (DICE), identifies clusters of patients who possess similar clinical profiles with respect to RVA using a novel optimization technique. [62] DICE is an outcome-aware clustering algorithm designed to cluster patients based on the severity and etiology of risk, specifically with respect to RVA outcomes. It employs a combination of autoencoders for representation learning, K-means clustering to identify patient subgroups, and logistic regression for outcome classification. The algorithm’s core lies in its joint optimization process, which targets representations from multidimensional data that lead to statistically significant outcome distributions among the clusters. This is achieved by optimizing a composite objective function, constrained by the statistical significance of the cluster-outcome associations, determined via a Wald test. The neural architecture search (NAS) component of DICE refines the process by selecting the optimal number of clusters and hyperparameters.

**Fig 5:**
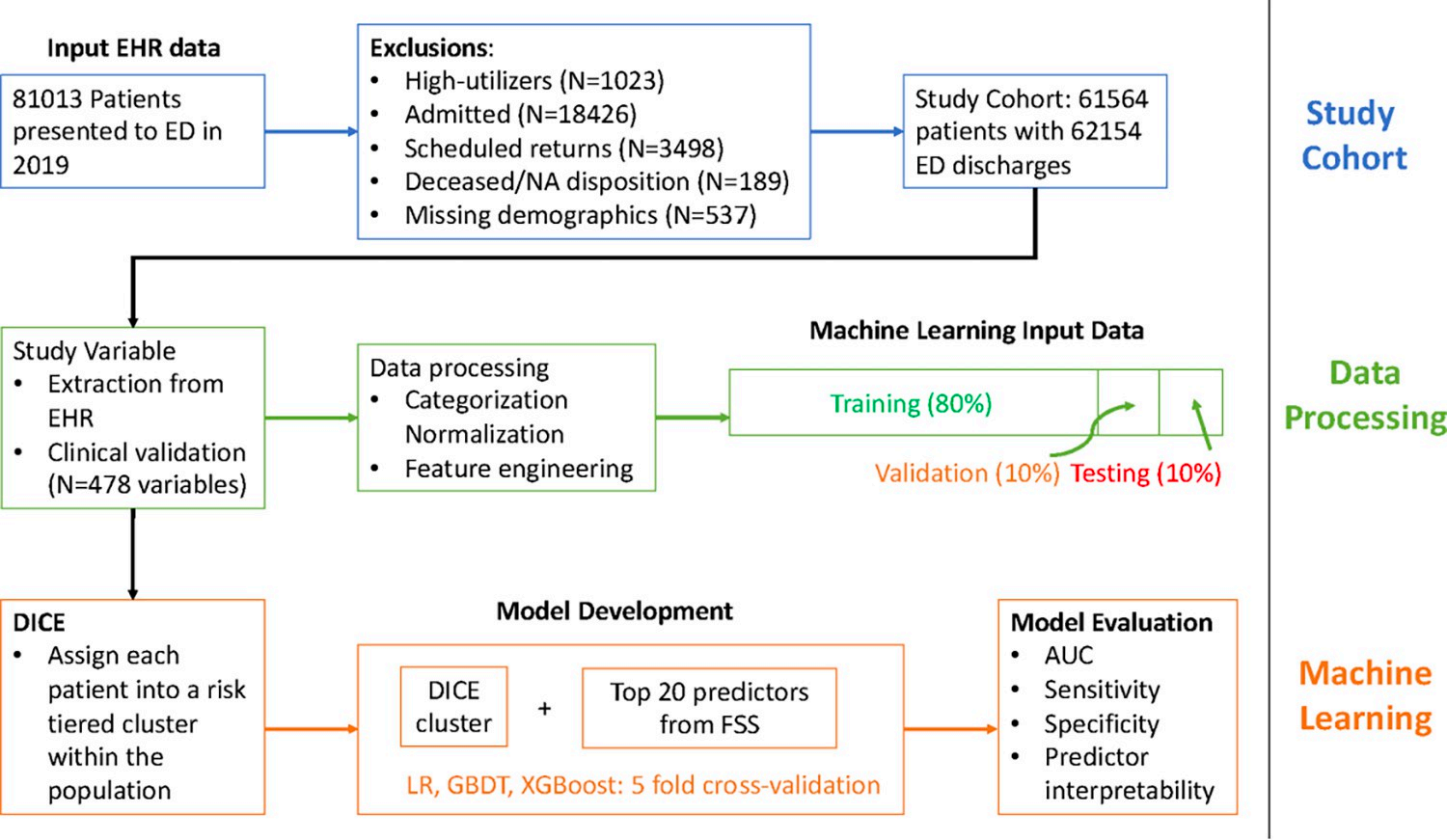
Modeling scheme. DICE: deep significance clustering; FFS: forward feature searches.

The modeling for the RVA prediction task consists of 2 steps: 1) the assignment of individual patients into risk-tiered clusters, followed by 2) classification using the cluster membership as a variable along with other selected predictors (Fig 5). In step 1, DICE was applied to study variables to stratify patients into risk-tiered clusters. In the 2^nd^ step, the cluster membership with different risk levels obtained from DICE are incorporated as input features in eXtreme Gradient Boosting (XGboost), Gradient Boosting Decision Tree (GBDT) and L1-regularized Logistic Regression (LR) for interpretable prediction. In addition to the cluster probabilities, to choose other predictors in these machine learning models, we used forward feature searches (FFS) for the best combination of features that returns the maximum AUC as model evaluation metric. Similar to Hong et al, [47] we chose top 20 variables using FFS aside from DICE cluster membership probabilities.

We conducted modeling with attention to the data imbalance. Since the frequencies of patients presenting to the ED vary each month, we split the dataset into training, validation, and testing sets with 8:1:1 balanced in each month of the study period. ED patient volume and clinical workflow are known to have seasonal effects. Thus, monthly stratification of data is to ensure that the test data appropriately capture the rare outcome given this seasonality. The test set was used to evaluate both DICE and the outcome prediction. Hyperparameters for XGboost, GBDT, LR, and DICE were optimized by maximizing the AUC of the validation set. Thresholds for all the classifiers were chosen according to the Youdens J statistic. We conducted experiments in the PyTorch framework on NVIDIA GeForce RTX 2070. Most predictive features for the 72-hr RVA are identified using SHapley Additive exPlanations (SHAP) values. [63] Hyperparameter settings are listed in Supplement. SHAP is used to explain the contributions of each variable to individual predictions by examining the impact of each variable’s value, such as large or small and 1 or 0, on the prediction direction, positive or negative, against a baseline variable value.

### Evaluation–quantitative

As a clinical baseline, we compared model performance against an ED RVA risk score developed by Gabayan et al. [4] This risk score is a 6-factor score using age, body mass index (BMI), systolic blood pressure (SBP), pulse, Charlson score, [64] and ED LOS trained using LR. BMI was omitted from our calculation due to the amount of missingness in the data. In addition, we applied a modified version of the RVA risk score using logistic regression coefficients fitted using our study data. For machine learning baseline, we tested classification approaches without DICE, and without FFS. Model evaluation metrics include area under the ROC curve (AUC), sensitivity, specificity, and positively likelihood ratio. S1 File describes the models and baselines experimented in this study, including the combination of methods (clustering, classification, and variable selection), as well as hyperparameter tuning.

Examining the clinically meaningful categories of patients for whom the model predicts at different accuracy levels will elucidate the clinical boundaries of the model’s performance and inform future implementation strategies. Thus, we conducted a sensitivity evaluation on the model’s performance as follows. We removed discharge-related variables from the input data and conducted the experiments with the same hyperparameters. Because older age is an identified risk factor related to RVA, [65] we also tested the model performance on patients 65 years old or above as a subgroup analysis. Furthermore, we tested the model performance on patients who had heart failure (HF), chronic obstructive pulmonary disease (COPD), urinary tract infection (UTI), and pneumonia as index ED discharge diagnoses coded by the ICD. The ICD definitions are listed in S2 File. Lastly, we evaluated the model performance across racial subgroups. AUCs are reported from the test sets. Definitions of diseases are listed in the S2 File.

### Evaluation–clinical

A team of three board-certified emergency physicians experienced in quality and patient safety case reviews conducted a clinical investigation of the classification results from the best performing model. The investigation aims to assess if the classification output aligns with clinical judgement, can be mapped to clinical sub-phenotypes of RVA, and can be interpreted with actionability. This process demonstrated the multifactorial nature of the RVA outcome and facilitated a comparative examination of model performance across different clinical scenarios. The clinical team reviewed 160 RVA cases from the development site EDs. These included 80 cases with the highest risk probabilities by the best-performing model (model-identified RVA), and 80 cases with lowest risk probabilities for RVA (model-missed RVA). The team reviewed all index and return ED visit data, hospital admission data, and prior relevant EHR data. Using an established RVA diagnosis classification system [66], RVA cases were organized into clinically relevant categories based on the index ED visit discharge diagnosis. The diagnosis categories include Cardiovascular, Pulmonology, Gastroenterology, Nephrology, Infection, General Medicine, Neurology, Oncology, Hematology, Rheumatology, Endocrinology, Surgery, Gynecology and Obstetrics, Otolaryngology. Ophthalmology, And Psychiatry. In addition, RVA cases were also classified by quality and patient safety root cause, utilizing a modified version of an established framework for quality and patient safety root-cause attribution of RVA.[26] The root causes include Illness-Related (progression of disease, failure of outpatient treatment, recurrent disease process, new problem related to index visit, complication), Physician-Related (admission indicated but consultant recommended outpatient management, failure of reassessment, misdiagnosis, treatment error, admission indicated on index visit and ED attending did not attempt to admit), Patient-Related (social issues, missed clinic follow-up, noncompliance), Healthcare System-Related (called back because of missed radiographic abnormalities, instructed to return for re-evaluation, sent from clinics, patient unable to get medication), and RVA unrelated to index visit. After each set of 20 cases were reviewed, the team met to review findings with the goal of expert consensus on the index ED visit discharge diagnosis and RVA root cause. Two primary reviewers assigned both an index visit diagnosis and quality and patient safety root-cause attribution for each RVA. In cases where a category assigned by the two physicians was divergent, a final decision was arbitrated by the third senior reviewer. Using Chi-Square tests with multiple testing correction, we tested the hypothesis of equal RVA prediction across diagnosis and root cause categories.

## Supporting information

S1 File Experimental setting. DICE: Deep significance clustering, LR: L1-regularized Logistic Regression, FSS: Forward feature searches, XGboost: eXtreme Gradient Boosting, GBDT: Gradient Boosting Decision Tree.
(DOCX)

S2 File Exclusion definition (ICD-10-CM). (DOCX)

S3 File ICD-DM codes definitions for pneumonia, COPD, UTI, and HF. (DOCX)
