# Supplementary material for: Aspiring to clinical significance: Insights from developing and evaluating a machine learning model to predict emergency department return visit admissions
Source: PLOS Digit Health. 2024 Sep 27;3(9):e0000606. doi: 10.1371/journal.pdig.0000606 (PMC11432862; doi:10.1371/journal.pdig.0000606)
Supplement: S1 File — DICE: Deep significance clustering, LR: L1-regularized Logistic Regression, FSS: Forward feature searches, XGboost: eXtreme Gradient Boosting, GBDT: Gradient Boosting Decision Tree. (DOCX) [file pdig.0000606.s001.docx]

**S1 File. Experimental setting. DICE: Deep significance clustering, LR: L1-regularized Logistic Regression, FSS: Forward feature searches, XGboost: eXtreme Gradient Boosting, GBDT: Gradient Boosting Decision Tree.**

| Model | Clustering | Classification | Variable selection | Hyperparameters |
| --- | --- | --- | --- | --- |
| DICE + GBDT | DICE | GBDT | N/A | number of clusters K = 2  n_estimators ~ U(1, 600, 50)  max_depth ~ U(1, 10)  min_samples_split ~ U(1, 10)  min_samples_leaf ~ U(1, 20)  max_features ~ U(5, 85, 5)  random_state = 88 |
| DICE + XGboost |  | XGBoost |  | number of clusters K = 2  max_depth ~ U[1, 18)]  gamma ~ U[1, 9]  reg_alpha ~ U[40, 180]  reg_lambda ~ U[0, 1]  colsample_bytree ~ U[0.5, 1]  min_child_weight ~ U(0, 10)  n_estimators ~ U(50, 2000, 50)  seed = 0 |
| DICE + LR |  | LR |  | C=[1e-6, 1e-5, 1e-4, 1e-3, 1e-2, 1e-1, 1e0, 1e1, 1e2, 1e3, 1e4, 1e5] |
| DICE + LR + FSS |  |  | FSS | number of clusters K = 2 |
| LR + FSS | N/A |  |  | C=[1e-6, 1e-5, 1e-4, 1e-3, 1e-2, 1e-1, 1e0, 1e1, 1e2, 1e3, 1e4, 1e5] |
| GBDT |  | GBDT | N/A | n_estimators ~ U(1, 600, 50)  max_depth ~ U(1, 10)  min_samples_split ~ U(1, 10)  min_samples_leaf ~ U(1, 20)  max_features ~ U(5, 85, 5)  random_state = 88 |
| XGBoost |  | XGBoost |  | max_depth ~ U[1, 18)]  gamma ~ U[1, 9]  reg_alpha ~ U[40, 180]  reg_lambda ~ U[0, 1]  colsample_bytree ~ U[0.5, 1]  min_child_weight ~ U(0, 10)  n_estimators ~ U(50, 2000, 50)  seed = 0 |
| LR |  | LR |  | C=[1e-6, 1e-5, 1e-4, 1e-3, 1e-2, 1e-1, 1e0, 1e1, 1e2, 1e3, 1e4, 1e5] |
| Baseline |  | Risk score |  | N/A |
| Modified baseline |  |  |  | N/A |

DICE: Deep significance clustering, LR: L1-regularized Logistic Regression, FSS: Forward feature searches, XGboost: eXtreme Gradient Boosting, GBDT: Gradient Boosting Decision Tree.
