# Supplementary material for: Aspiring to clinical significance: Insights from developing and evaluating a machine learning model to predict emergency department return visit admissions
Source: PLOS Digit Health. 2024 Sep 27;3(9):e0000606. doi: 10.1371/journal.pdig.0000606 (PMC11432862; doi:10.1371/journal.pdig.0000606)
Supplement: S2 File — (DOCX) [file pdig.0000606.s002.docx]

**S2 File. Exclusion definition (ICD-10-CM)**

Laceration: S**, W54.**, W55.**, Y04.1XXA, S41.159A, S41.152A, S41.151A, S41.159A

Abscess: L02.**
