# Supplementary material for: Aspiring to clinical significance: Insights from developing and evaluating a machine learning model to predict emergency department return visit admissions
Source: PLOS Digit Health. 2024 Sep 27;3(9):e0000606. doi: 10.1371/journal.pdig.0000606 (PMC11432862; doi:10.1371/journal.pdig.0000606)
Supplement: S3 File — (DOCX) [file pdig.0000606.s003.docx]

**S3 File. ICD-DM codes definitions for pneumonia, COPD, UTI, and HF.**

Pneumonia: B59.*, J69.*, J09.*- J18.*

COPD: J44*, J43.9

UTI: N10.*-12.*, O23.*, N39.0

HF: I50.*, I11.0
